# The TOP vector: a new high-titer lentiviral construct for delivery of sgRNAs and transgenes to primary T cells

**DOI:** 10.1016/j.omtm.2020.10.020

**Published:** 2020-10-27

**Authors:** Daryl Humes, Stephanie Rainwater, Julie Overbaugh

**Affiliations:** 1 Division of Human Biology, Fred Hutchinson Cancer Research Center, Seattle, WA, USA

**Keywords:** gene therapy, primary T-cells, lentiviral vector, chimeric antigen receptor, CRISPR Cas9, gene editing

## Abstract

Efficient delivery of nucleic acids for the engineering of primary T cells is central to the study of the basic biology of these key immune effector cells and has clinical implications. To date, lentiviral vectors delivering guide RNAs for CRISPR-Cas9 editing are not optimal for use in primary cells. Herein, we describe the T cell optimized for packaging (TOP) vector for delivering guide RNAs and transgenes into primary T cells. The TOP vector produces high-titer virus compared to a routinely used guide RNA vector, resulting in a ~10-fold increase in transduction in T cells. Moreover, a TOP vector expressing a chimeric antigen receptor and a guide RNA targeting the T cell receptor showed an ~5- to 9-fold increased transduction efficiency with ~2- to 3-fold higher expression compared to the commonly used epHIV7 vector and was simultaneously able to mediate efficient knockout of the endogenous T cell receptor in >71% of transduced cells upon Cas9 electroporation. The increased packaging of the TOP vector genome into viral particles appears to contribute to its higher transduction efficiency. The TOP vector represents an optimal tool for tandem delivery of transgenes and guide RNAs to primary T cells for use in functional screens and immunotherapy applications.

## Graphical Abstract

**Figure:**
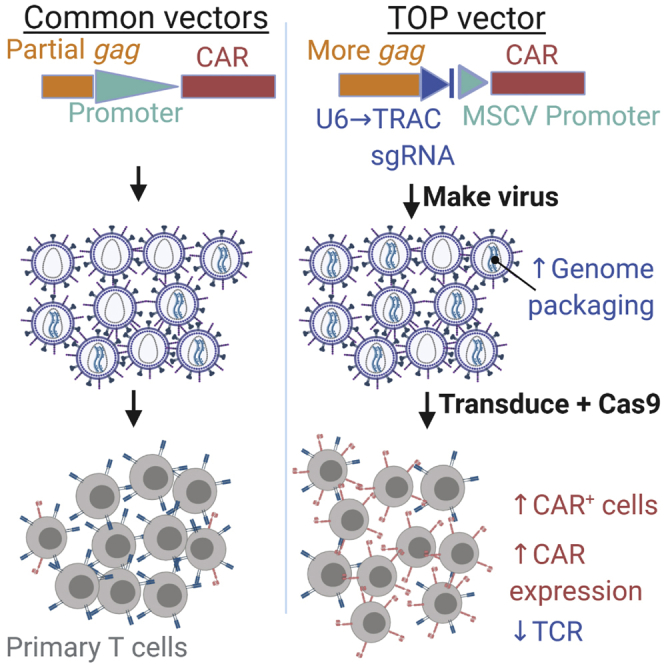

## Teaser

The T cell optimized for packaging (TOP) vector is a lentiviral construct for efficient dual delivery of guide RNAs and transgenes into primary T cells. The TOP vector provides a useful tool for CRISPR screening and potential immunotherapy applications such as delivering chimeric antigen receptor while depleting endogenous T cell receptor expression.

## Introduction

Gene engineering is central to both the transfer of specific genes or gene fragments and directed editing of specific gene targets for the purposes of modifying cell function. Primary T cells represent a key potential target for gene engineering approaches because they can be modified and expanded *ex vivo* prior to re-introduction into a patient. This approach is fundamental to cutting edge immunotherapy approaches to promote T cell activity against specific immunological targets, including the introduction of combined antigen recognition and signaling molecules such as the chimeric antigen receptor (CAR). Primary T cells are also of high interest as key regulatory and effector cells of the immune system, and they are the major target for HIV infection. Thus, methods that can improve gene engineering in primary T cells are valuable for a range of biological studies and clinical applications.

Gene editing through CRISPR-Cas9 has revolutionized biomedical science, both because of its utility in dissecting biological pathways and, more recently, for its application to clinical medicine, as exemplified by the newest approaches in cancer immunotherapy.^1^ It relies on introducing guide RNAs designed to a specific genetic locus into the cell that results in precision editing of that gene target mediated by the Cas9 protein (or derivatives thereof). CRISPR-Cas9 editing can be applied broadly across a collection of guide RNAs to screen for genes that regulate a phenotype of interest. The use of CRISPR-Cas9 technology for high-throughput gene knockout screens is now well established for use in cell lines, but is much less widespread in primary T cells.^2^^,^^3^ One major bottleneck in performing such CRISPR-Cas9 screens in primary cells is the relative inefficiency of constructs delivering the guide RNAs to primary cells.

Common gene engineering approaches typically rely on a lentiviral vector for the delivery of a specific gene, coding sequence, or single-guide RNA (sgRNA) into cells. Because lentiviral vectors are capable of integrating their genomes into the host cellular DNA, the sequence encoded within them can be expressed as transgenes. Lentiviral vectors that have been described and used so far for introducing guide RNAs required for CRISPR-Cas9-mediated gene knockout into primary T cells have not been optimized for production or for transgene expression in primary T cells.^2^^,^^3^ The T cell optimized for packaging (TOP) vector described herein uses basic aspects of lentiviral vector design with significant modifications to optimize sequence delivery into primary T cells. The TOP vector allows for the preparation of high-titer lentiviral vectors that lead to efficient introduction of guide RNAs and high expression of transgenes in primary T cells, and it can be used to perform both editing and transgene expression functions simultaneously upon electroporation with Cas9. Thus, the TOP vector represents a formidable tool for producing high-titer lentivirus for introducing guide RNAs into primary T cells in conjunction with high-level sustained expression of a paired transgene. This will facilitate high-throughput CRISPR-Cas9 screens in primary cells either alone, or when paired with reporter transgenes, or potential gene therapy applications when paired with transgenes and guide RNAs against targets of interest, for example, knocking out of the endogenous T cell receptor (TCR) while simultaneously expressing a CAR.^4^

## Results

### Development of the TOP vector for expression of mCherry and sgRNA in primary CD4^+^ T cells

In order to design an sgRNA-expressing lentivirus with increased transduction efficiency in primary CD4^+^ T cells, a number of modifications were made to lentiGuide.mCherry,^5^ an mCherry-expressing vector that represents a commonly used pRRL.SIN^6^ vector architecture for delivery of sgRNA to mediate editing by CRISPR-Cas9. First, the central polypurine tract/central termination sequence (cPPT/CTS) were moved upstream of the U6-sgRNA expression cassette because a previous report had suggested that locating the cPPT/CTS in this manner can serve to increase the lentiviral titer of vectors encoding sgRNA guides.^7^ In so doing, the simian virus 40 (SV40) polyadenylation signal was replaced with a bovine growth hormone (BGH) polyadenylation signal, and the promoter driving expression of the vector genomic transcript in producer cells was changed from the Rous sarcoma virus (RSV) enhancer/promoter to a cytomegalovirus (CMV) enhancer/promoter, which may also serve to increase titer.^8^ Additionally, the full-length human elongation factor 1ɑ (EF1-α) promoter was replaced with the mouse stem cell virus (MSCV) promoter, which is much shorter and has been shown to perform very well in primary T cells.^9^ These changes were focused on improving transcription of the vector genomic RNA in producer cells and transgene expression in transduced cells.

**Figure 1:**
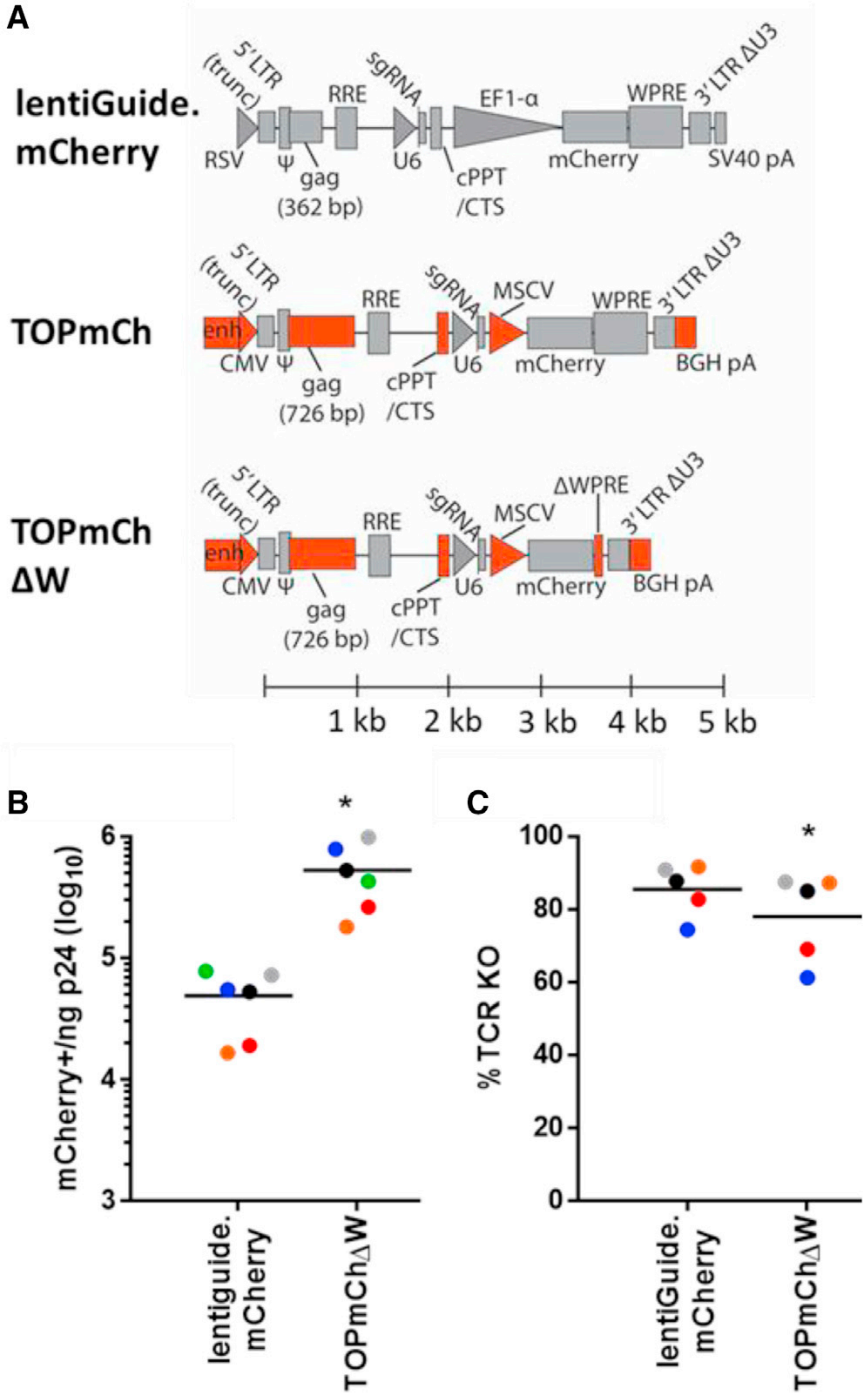
Comparison of TOPmChΔW and lentiGuide.mCherry genome structure and performance (A) Maps comparing the TOP and lentiGuide.mCherry genome architectures. Red coloring indicates those elements in the TOP vector that differ from lentiGuide.mCherry; gray indicates the common sequences. Maps are lined up according to the ruler at the bottom of the figure such that the first transcribed nucleotide of the vector genome is at the origin. LTR, long terminal repeat; RSV, Rous sarcoma virus promoter; trunc, indicates that the element is truncated; Ψ, packaging signal; RRE, Rev response element; sgRNA, single-guide RNA; U6, U6 type III polymerase promoter; cPPT/CTS, central polypurine tract/central termination sequence; EF1-α, human elongation factor 1α promoter; WPRE, woodchuck hepatitis virus post-transcriptional response element; SV40 pA, simian virus 40 polyadenylation signal; CMV, cytomegalovirus promoter; enh, CMV enhancer; MSCV, mouse stem cell virus promoter; BGH pA, bovine growth hormone polyadenylation signal. (B) A comparison in the transduction efficiency between TOPmChΔW and lentiGuide.mCherry. The number of mCherry-positive cells normalized to the ng of p24 are shown. Results are from CD4^+^ T cells from six donors indicated by the different colored points with the black line indicating the average value. ∗p < 0.05 as determined by a paired two-tailed t test comparing TOPmChΔW to lentiGuide.mCherry. (C) Comparison of TCR inactivation by TOPmChΔW and lentiGuide.mCherry. The percentage of transduced cells knocked out for TCR expression (% TCR KO) was determined by the loss of CD3 expression in mCherry-positive cells using flow cytometry. Results from CD4^+^ T cells from five donors are shown, indicated by the different colored points with the black line indicating the average value. The cells edited in this experiment are from five of the six donors transduced in B). ∗p < 0.05, as determined by a paired two-tailed t test.

To attempt to further increase virus titer, we focused on increasing the packaging efficiency of these vector genomes. To do so, the amount of *gag* sequence overlapping the *psi* packaging signal was increased from 362 to 726 bp, because a longer portion of the *gag* sequence has been reported to increase the *trans*-packaging of lentiviral genomes by the HIV-1 Gag protein.^10^^,^^11^ Importantly, the extended *gag* sequence in the TOP vector maintains a 2-bp frameshifting insert early in the *gag* sequence that is designed to eliminate Gag protein expression from the construct. We designated this collection of changes as the TOP vector architecture, and called this initial mCherry-expressing construct TOPmCh (Figure 1A).

Because it has been suggested that CG-dinucleotide-rich transcripts are subject to degradation by the ZAP protein, which may affect viral titer,^12^^,^^13^ a variant of the TOP vector called TOPmChdW, in which the CG-rich woodchuck hepatitis virus post-transcriptional regulatory element (WPRE) sequence was mostly removed, was also constructed. Of note, despite the addition of the extra *gag* sequence, the TOPmChΔW genome is smaller (approximately 4.1 kb) compared to the lentiGuide.mCherry vector (4.9 kb), primarily due to the difference in size of the EF1-α promoter and the MSCV promoter. In order to compare both transduction and gene editing efficiency, an sgRNA targeting the TCR α chain (*TRAC*) locus^4^ was cloned into the TOPmChΔW vector and the lentiGuide.mCherry vector. Viruses were prepared in tandem, and equivalent amounts of virus based on p24 Gag protein levels were used to transduce T cells from two different donors. The TOPmChΔW had a substantial (p = 0.01) 10.8-fold increase in average titer (5.3 × 10^5^ mCherry-positive cells/ng p24) as compared to lentiGuide.mCherry (4.9 × 10^4^ mCherry-positive cells/ng p24; Figure 1B). Editing efficiency mediated by the different vectors was determined by expression of sgRNA from transduced TOPmChΔW vector or lentiGuide.mCherry and electroporation of the Cas9 protein complexed with a non-targeting sgRNA for optimal editing efficiency,^3^ and the levels of editing were determined by staining for the TCR complex on the cell surface. The TOPmChΔW vector mediated knockout of the TCR with slightly less efficiency in transduced primary CD4^+^ T cells (1.1-fold; 78.0% versus 85.4%; Figure 1C) that were transduced with saturating amounts of vector. The differences, although subtle, were statistically significant (p = 0.038) when performing paired comparisons across cells from five donors (Figure 1C). Despite the higher transduction efficiency, we suspect that pairing Cas9 expression with sgRNA expression in the same lentiviral vector will continue to be a problem in primary CD4^+^ T cells in the TOP vector. Consistent with previous studies,^14^ we saw very little expression of Cas9 in primary CD4^+^ T cells, despite robust expression in the THP-1 cell line. This was true even when Cas9 expression was driven by the MSCV promoter used in the TOP vector (Figure S1).

### Comparison of transduction efficiency and CAR expression using the TOP and epHIV7 vectors

**Figure 2:**
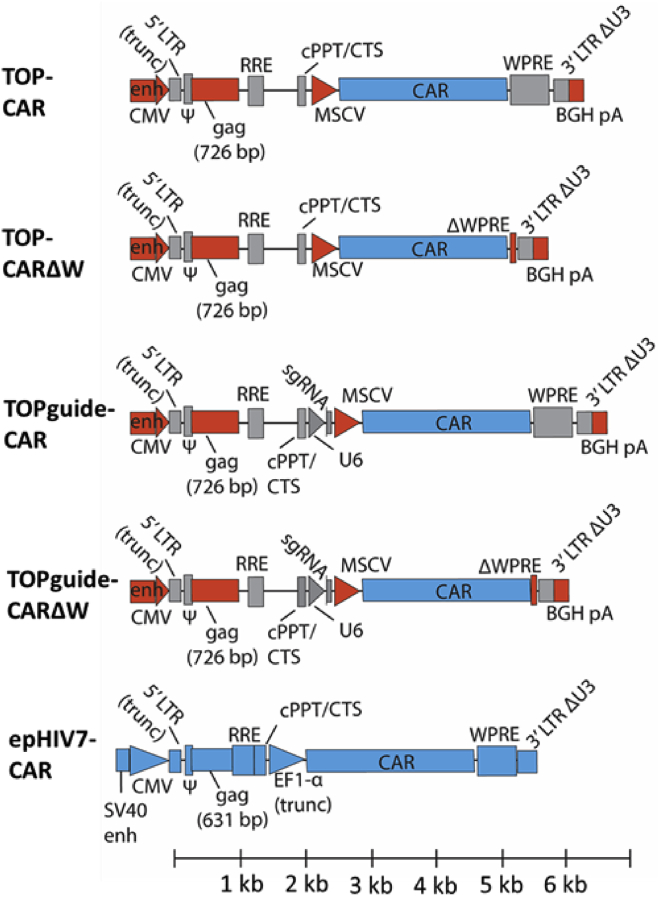
Comparison of genome structure of TOP vector derivatives expressing a CAR and the epHIV7 vector Maps showing different TOP vectors expressing a CAR in comparison to the epHIV7 vector. Elements colored in red in the TOP vectors highlight modifications made to the vector; elements in gray are common with lentiGuide.mCherry (Figure 1A) and elements in blue are from the epHIV7-CAR vector. Maps are lined up according to the ruler at the bottom of the figure such that the first transcribed nucleotide of the vector genome is at the origin. SV40 enh, simian virus 40 enhancer; CAR, chimeric antigen receptor; all other acronyms and short forms are as in Figure 1A.

Given the clinical importance of being able to efficiently deliver CARs to T cells, we next sought to examine the performance of the TOP vector when expressing a CAR. To be able to easily monitor CAR expression, a previously described CAR containing a streptavidin II tag^15^ was cloned into the TOP vector. Four different configurations were generated to examine the effects of the U6-sgRNA element and WPRE on transduction efficiency and CAR expression (Figure 2). These included the following: (1) TOP-CAR, without a U6-sgRNA cassette but with an intact WPRE; (2) TOP-CARΔW, without a U6-sgRNA cassette and with most of the WPRE removed; (3) TOPguide-CAR, with an sgRNA targeting the endogenous TRAC and an intact WPRE; and (4) TOPguide-CARΔW, with an sgRNA targeting the endogenous TRAC and with most of the WPRE removed. The insert sizes of these vectors ranged from approximately 5.6 kb for TOP-CARΔW to 6.5 kb for TOPguide-CAR. The performance of the TOP vectors was gauged by comparison to the commonly used epHIV7 vector.^16^^,^^17^ The epHIV7 vector differs from the TOP vector genome structure in a number of ways, including having a truncated EF1-α promoter driving CAR expression, absence of a polyadenylation signal after the 3′ long terminal repeat (LTR), less *gag* content (631 bp versus 726 bp for TOP vectors), and an approximate insert size of 5.5 kb (Figure 2).

**Figure 3:**
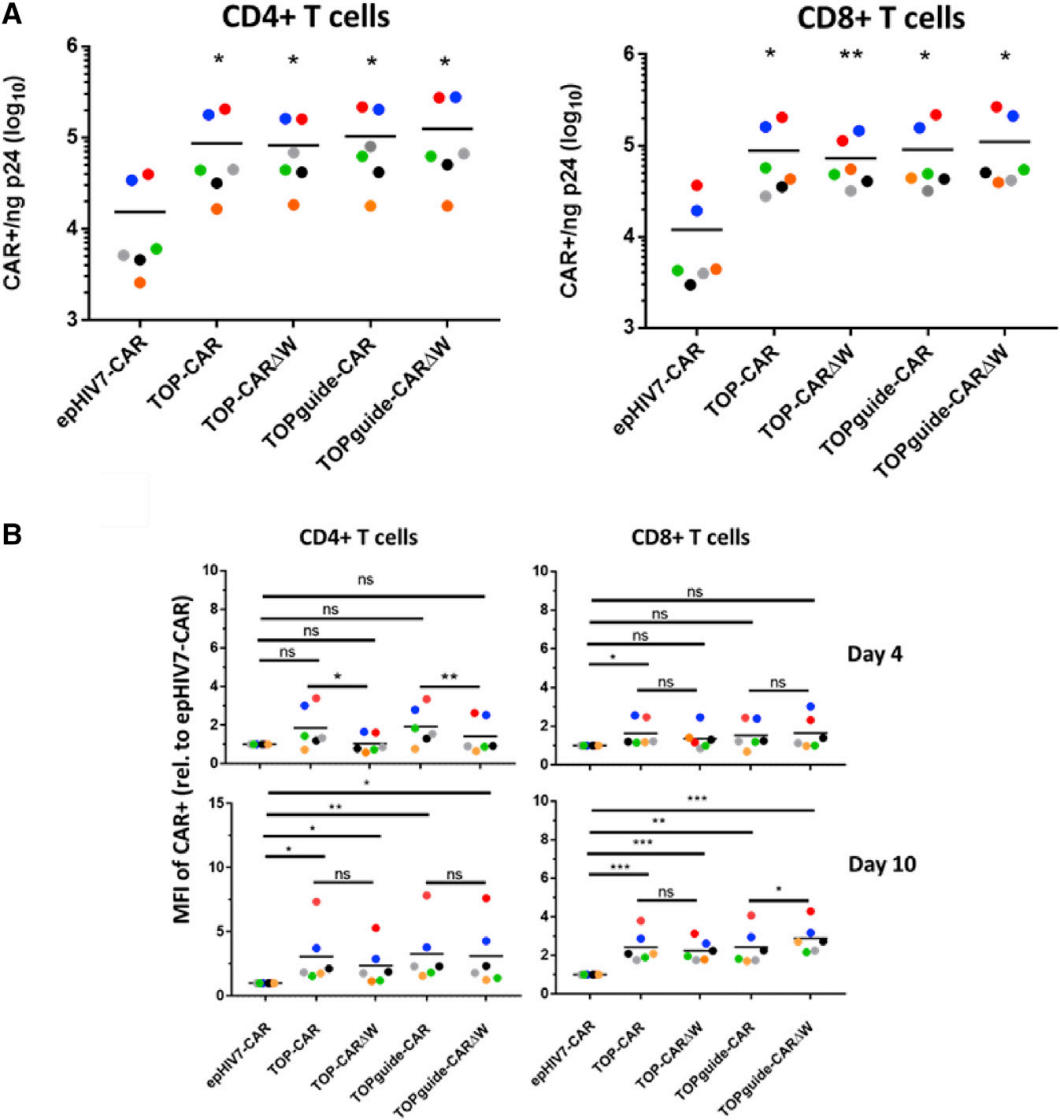
Comparison in the transduction efficiency and CAR expression between TOP vectors and epHIV7 (A) Transduction efficiency of streptavidin-tagged CAR expressing vectors in CD4^+^ and CD8^+^ T cells from six donors is indicated by the different colored points. The number of cells expressing a streptavidin-tagged CAR^+^ per ng of p24 determined at 4 days post-transduction is shown. ∗p < 0.05, ∗∗p < 0.01 as determined by a paired two-tailed t test comparing the different vectors to epHIV7. (B) Relative mean fluorescence intensity (MFI) of CAR-positive cells transduced in (A) at 4 and 10 days post-transduction. Results from six donors are shown as indicated by the different colored points, with the black line indicating the average value. Data are derived from cells transduced with 20 ng of p24 per million cells in the case of epHIV7-CAR, and 2 ng of p24 per million cells for all other vectors. All data are normalized to the epHIV7-CAR MFI to account for the fact that MFI data from different donors and time points were obtained on different days. Statistical comparisons between epHIV7-CAR and all other vectors were performed using a ratio paired t test, and statistical comparisons between paired vectors with and without the WPRE were performed using a paired two-tailed t test. ∗p < 0.05, ∗∗p < 0.01, ∗∗∗p < 0.005. ns, not significant.

Viruses prepared with the various TOP vectors had similar trends for transduction efficiency and CAR expression in six different donors (Figure 3). Transduction efficiencies ranged from an average of 1.7 × 10^4^ to 2.7 × 10^5^ CAR-positive cells per ng of p24 in CD4^+^ T cells and 2.8 × 10^4^ to 2.4 × 10^5^ CAR-expressing cells per ng of p24 in CD8^+^ T cells at 4 days post-transduction. The TOPguide vectors had comparable or higher transduction efficiency than TOP vectors lacking the U6-sgRNA cassette (Figure 3A), suggesting that addition of the U6-sgRNA cassette does not decrease transduction efficiency in this setting. Overall transduction efficiencies were increased by 5.4- to 8.1-fold in CD4^+^ T cells and were 6.1- to 9.3-fold higher in CD8^+^ T cells as compared to epHIV7 (Figure 3A), and all increases in transduction when comparing the different TOP vectors to epHIV7 were statistically significant when comparing across the different donors (Figure 3A).

Expression of the CAR in TOP vectors was qualitatively higher than epHIV-7 when 20 ng of p24 per million cells of each vector was transduced, as exemplified by sample flow cytometry plots (Figure S3). Importantly, when controlling for vector saturation and comparing transductions with 2 ng of p24 of TOP vector to 20 ng of p24 of epHIV7, the average level of expression of the CAR in the TOP vector as determined by mean fluorescence intensity in six donors was at least as high or higher, by as much as 1.9-fold at 4 days post-transduction in CD4^+^ T cells and by as much as 1.6-fold higher in CD8^+^ T cells when compared to the epHIV7 vector (Figure 3B). These differences were more marked when CAR expression was assayed at 10 days post-transduction, with CAR expression increases of 2.4- to 3.3-fold in CD4^+^ T cells and 2.2- to 2.9-fold in CD8^+^ T cells. At 10 days post-transduction, there were modest decreases of 1.3- and 1.1-fold in CAR expression in CD4^+^ T cells and CD8^+^ T cells when comparing TOP-CARΔW, which lacks an intact WPRE, to the TOP-CAR construct. Interestingly, the addition of the U6-sgRNA cassette seemed to abrogate these modest effects, with the TOPguide-CARΔW vector even displaying a statistically significant 1.2-fold higher expression at 10 days post-transduction in CD8^+^ T cells (Figure 3B).

### Simultaneous CAR expression and endogenous TCR knockout using the TOP vector

**Figure 4:**
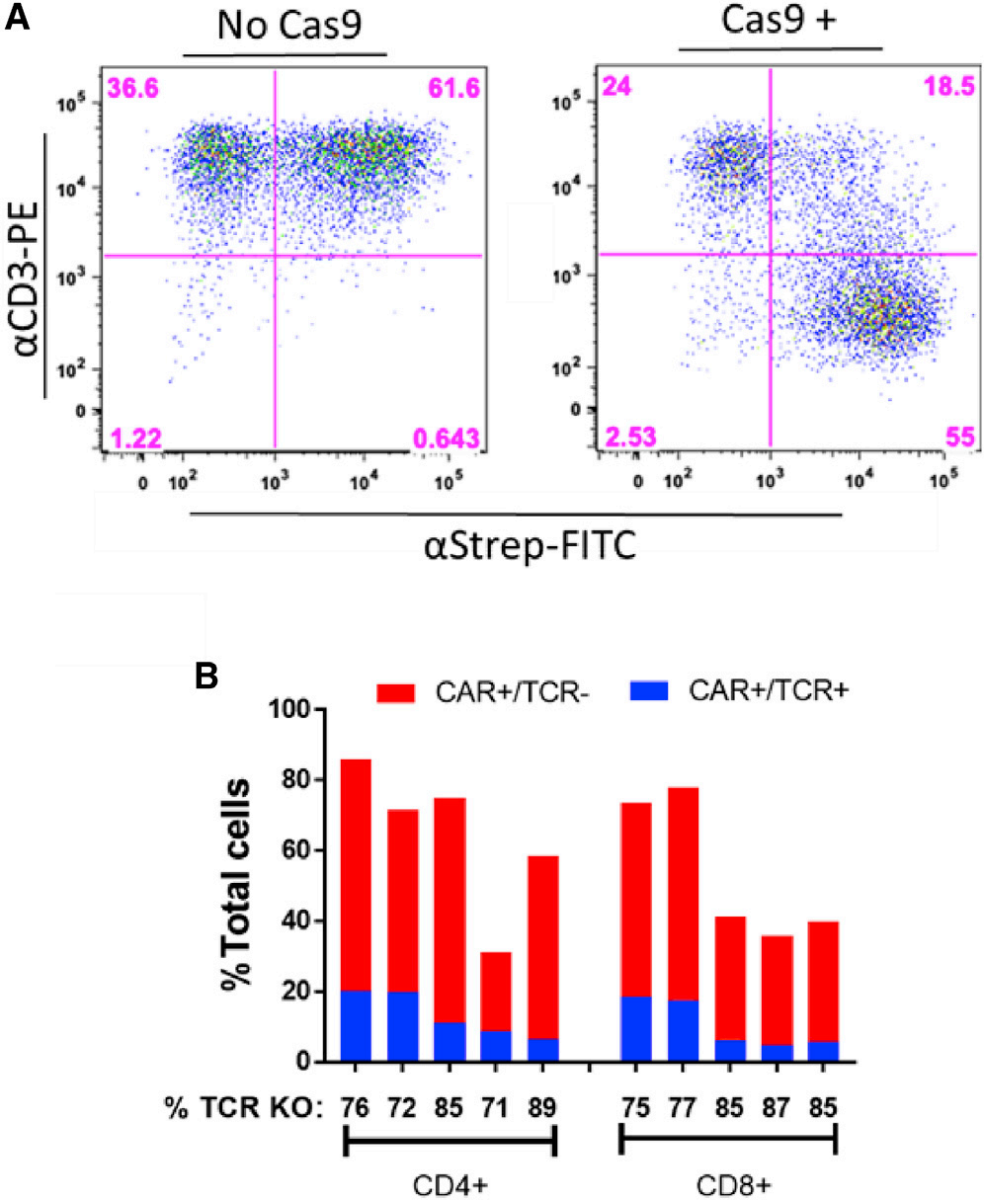
TCR knockout in TOPguide-CARΔW-transduced cells TCR knockout was determined by the loss of expression of CD3 on the cell surface of CAR-positive cells. (A) Shows an example of a flow cytometry plot for CD8^+^ T cells from donor 2. CAR expression is shown by staining with the anti-streptavidin-FITC antibody on the x axis, and TCR expression is shown by staining with an anti-CD3-PE antibody on the y axis. (B) Aggregate of editing experiments in CD4^+^ and CD8^+^ T cells from five different donors. Each bar represents a single donor, with the red portions of the bar representing transduced cells for which TCR expression was lost (CAR^+^/TCR^−^ cells, lower right quadrant in A), and the blue portions representing transduced cells that maintained TCR expression (CAR^+^/TCR^+^ cells, upper right quadrant in A). The percentage of TCR knockout (% TCR KO) is shown under each donor and was derived from calculating the percentage of CAR^+^ cells for which TCR expression was lost.

To test the editing efficiency of a TOP vector co-expressing a CAR and a sgRNA, a saturating amount of TOPguide-CARΔW, which includes an sgRNA to target the TRAC locus (Figure 2), was used to transduce CD4^+^ and CD8^+^ T cells from five different donors. The cells were electroporated with Cas9 non-targeting control sgRNA ribonucleoprotein complexes, and the levels of editing were determined by staining for the TCR complex on the cell surface (Figure 4A). The transduction efficiencies and editing efficiencies in the different T cell subsets from two different donors are summarized in Figure 4B. The transduction efficiencies in these experiments were variable, ranging from 31.1% to 85.9% for CD4^+^ T cells, resulting in anywhere from 22.2% to 65.7% of the total cells being both CAR-positive and TCR-negative after transduction and electroporation. The overall editing efficiency between donors was much more consistent, averaging 79% and ranging from 71% to 89% in CD4^+^ T cells. Results in CD8^+^ T cells were very similar, with a variable range of transduction resulting in anywhere from 31.2% to 60.3% of the cells being CAR-positive and TCR-negative after electroporation. Overall editing efficiency was consistently high, ranging from 75% to 87% and averaging 82% editing.

### Increased packaging of transcripts from TOP vector constructs

**Figure 5:**
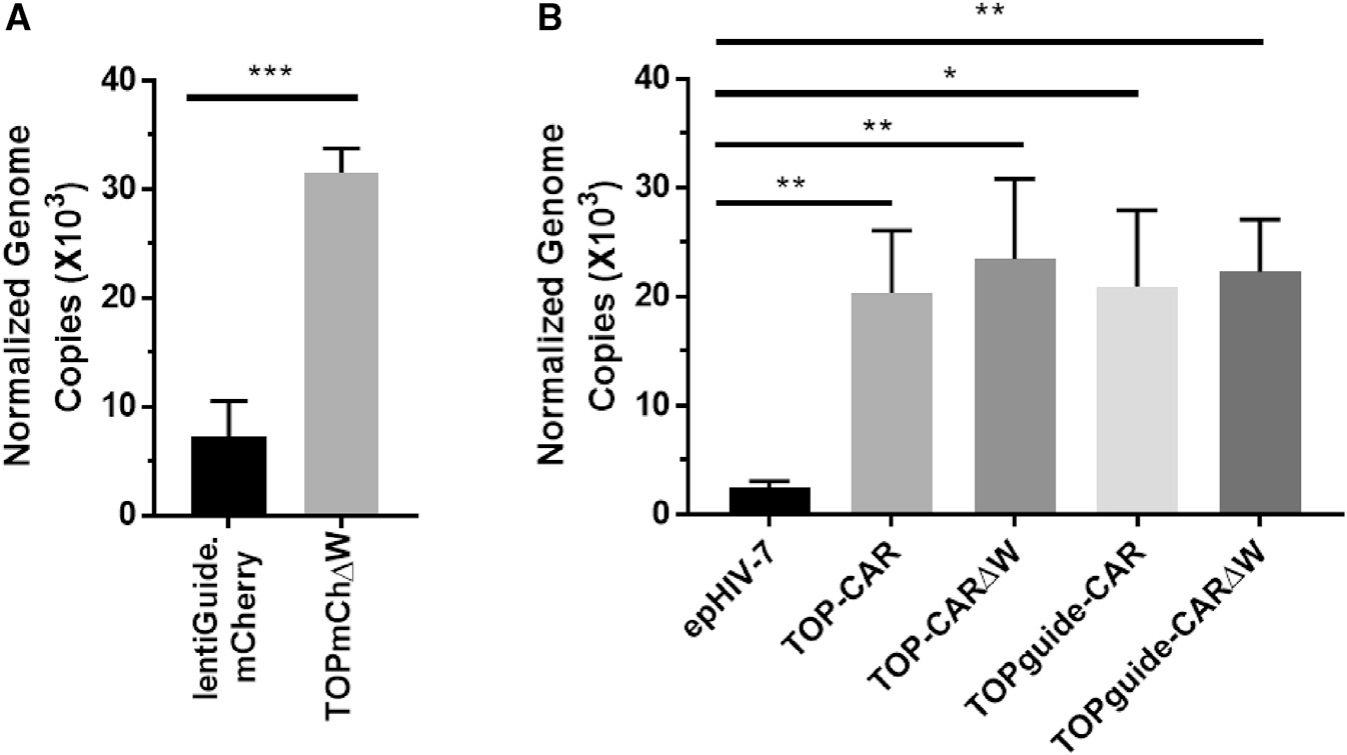
Comparison of genome packaging of different vectors Normalized genome copies were calculated by dividing the number of vector copies per μL of cDNA as determined by ddPCR by the p24 concentration (in ng/mL). Error bars represent the standard deviation from three virus stock replicates. ∗p < 0.02, ∗∗p < 0.01, ∗∗∗p < 0.001 as determined by an unpaired non-parametric t test. (A) Comparison between lentiGuide.mCherry and TOPmChΔW. (B) Comparison between TOP-CAR, TOP-CARΔW, TOPguide-CAR, TOPguide-CARΔW, and epHIV7-CAR.

There are a number of modifications in the TOP vector genome meant to improve expression, titer, and overall transduction efficiency. The most novel modification, the inclusion of an additional HIV-1 *gag* sequence, was included primarily as an attempt to increase genome packaging efficiency. To assess the relative packaging efficiency of genomes transcribed by the TOP vector compared to lentiGuide.mCherry and epHIV7, we compared the relative packaging efficiency of the different constructs. To do this, the genome copies of each virus were determined and normalized to the p24 concentration, thus giving an estimate of the number of genomes packaged in a given number of viral particles. The p24 concentrations were similar and not significantly different between TOP vectors and other viral stocks, suggesting similar levels of viral particle production (data not shown). Virus made with the TOPmChΔW vector had a ~4.4-fold increase (p < 0.001) in normalized genome copies (vector cDNA/p24 concentration) as compared to lentiGuide.mCherry, indicating that more viral genomes were present in the viral supernatant (Figure 5A). Interestingly, the difference in viral packaging was even more striking when comparing the TOP-CARΔW vector to epHIV7, with increased packaging ranging from 8.4- to 9.7-fold for TOP-CAR and TOP-CARΔW, respectively (p < 0.01; Figure 5B). These data suggest that increased *trans*-packaging of the vector genomes in viral particles contributes substantially to the increased infectivity of the TOP vectors.

## Discussion

This report details the development of the TOP lentiviral genome for use in the transduction of primary T cells. The TOP vector has substantially increased transduction efficiency as compared to other commonly used lentiviral vectors in primary T cells, whether expressing a reporter protein or other transgenes, such as CARs. Moreover, the TOP vector has the versatility to pair transgene expression with CRISPR-Cas9-mediated gene knockout when Cas9 is introduced by electroporation, making it valuable for applying these dual approaches to engineer cells *ex vivo* with desirable properties. As an example of this, we demonstrated the ability to express high levels of CAR while simultaneously knocking out endogenous TCR expression.^18^ The broad applicability of the TOP vector to other therapeutically desirable transgene and knockout targets can only be inferred, as no other targets were assayed in this study.

The TOP vector design incorporates various modifications to increase titer in primary T cells, most notably the use of the MSCV promoter, which is known to be a strong promoter in primary T cells.^9^ The TOP vector design also leverages results of previous studies showing that including more HIV-1 *gag* RNA sequence after the *psi* packaging signal results in increased *trans*-packaging of vector RNA by HIV-1 particles.^10^^,^^11^ More commonly used vectors, such as those belonging to the pRRL.SIN family^6^ or pHIV7 family,^17^ encode suboptimal amounts of HIV-1 *gag* compared to the 726 nt encoded by the TOP vector.^10^ As a result, there was a substantial boost in packaging of TOP vector genomes compared to TOP vector derivatives. This increase in packaging was especially striking when comparing the TOPguide-CAR vector to the epHIV7 vector expressing a CAR. This is most likely not due to the trivial explanations of increased genome production in the producer cells or more efficient packaging of shorter vector genomes because transcription of the genomes of both vectors is driven by similar CMV promoters, and the RNA genome expressed by TOPguide-CAR vector is approximately 1 kb larger than that expressed from the epHIV7-CAR vector. These data suggest that the increased packaging imparted is a key driver of the net increase in viral titer; however, the individual contribution of each modification to this specific phenotype and whether they completely synergize to increase viral titer were not explicitly tested.

Another modification that was made to increase titer was gleaned from the development of the widely used lentiCRISPRv2 vector, in which it was found that moving the cPPT/CTS before the U6-sgRNA cassette increased the titer of the vector.^7^ Although the mechanism by which this happens is unclear, it is possible that, much like what has been suggested with increased *gag* content,^10^^,^^11^ moving the native HIV-1 sequence closer to the packaging elements may serve to further insulate the critical RNA secondary structure from potential interference with downstream transgenes or other non-HIV-1 elements.

The WPRE is well known to increase transgene expression,^19^ although its utility may be context-dependent.^20^ However, we posited that the CG dinucleotide-rich WPRE could also serve to decrease transduction efficiency because it is a potential target for ZAP degradation.^12^^,^^13^ Ultimately, removing the WPRE did not result in increased transduction efficiencies in CAR constructs, and it modestly decreased transgene expression unless the U6-sgRNA cassette was present, which was particularly apparent in CD8^+^ T cells (Figure 4B). How the presence of the U6-sgRNA cassette might be compensating for the loss of the WPRE is unclear; however, the U6 promoter, which is normally transcribed by type III RNA polymerase, has been shown to act as a PolII promoter as well.^21^ One could speculate that the U6 promoter may be recruiting common factors that also boost transcription from the MSCV promoter to offset the absence of the WPRE. This is a notable finding, as it suggests that in certain contexts the WPRE may be dispensable for optimal expression when a transgene is paired with the U6 promoter, thus allowing for the expression of larger transgenes in the vector.

In summary, by combining a variety of modifications, we have developed the TOP vector for optimal transgene expression and gene editing in primary T cells. The TOP vector represents a useful tool for gene editing experiments in primary cells as well as having potential in the realm of immunotherapy or related clinical applications.

## Materials and methods

### Plasmids and cloning

pAW13.lentiGuide.mCherry^5^ (Addgene #104375; herein called lentiGuide.mCherry) and pLentiCRISPRv2-mCherry, which is based on lentiCRISPRv2,^7^ but expresses mCherry in place of puromycin resistance (Addgene #99154), were used as template vectors on which to build a TOP vector expressing mCherry (TOPmCh). Briefly, the core EF1-α promoter-driven *Streptococcus pyogenes* (Sp)Cas9-mCherry fusion protein in lentiCRISPRv2-mCherry was replaced with the full EF1-α promoter-driven mCherry cassette from lentiGuide.mCherry. As a result, the orientation of the cPPT/CTS relative to the U6-sgRNA cassette differs from the lentiGuide.mCherry backbone, and the vector now includes a BGH polyadenylation signal after the 3′ LTR and a CMV promoter/enhancer in place of the RSV promoter/enhancer to drive transcript expression in producer cells.

The full-length EF1-ɑ promoter was subsequently replaced with a synthesized MSCV promoter^22^ (based on the U3 sequence from GenBank: M17246.1) by cloning into the EcoRI and BsiWI restriction sites. The TOPmCh vector genome was finalized by extending the HIV-1 *gag* sequence from 362 to 726 bp. Because, the first 362 bp of *gag* sequence of lentiGuide.mCherry most closely match with the LAI strain (GenBank: A04321), the HIV *gag* sequence from nucleotide 363 to 726 of the LAI strain were PCR amplified and Gibson cloned directly upstream of the Rev response element (RRE). Notably, the TOPmCh vector and derivatives maintain a 2-nt frameshifting insertion at position 45 of the *gag* sequence.

A derivative called TOPmChΔW was created in which all but the final 85 bp of the WPRE sequence was removed by digestion of the MluI and SacII restriction sites, generating blunt ends with a Klenow fragment (New England Biolabs, Ipswich, MA, USA), and re-ligating. A map comparing the TOPmCherry and TOPmChΔW architecture to the lentiGuide.mCherry architecture is shown in Figure 1A.

The TOPguide-CAR and TOPguide-CARΔW vectors were generated by PCR amplifying a streptavidin-tagged CAR,^15^ and cloning in place of the mCherry sequence in TOPmCh and TOPmChΔW. The TOP-CAR and TOP-CARΔW vectors were made by excising the U6-sgRNA cassette at flanking KpnI and EcoRI sites, generating blunt ends with Klenow fragment treatment (New England Biolabs, Ipswich, MA, USA), and re-ligating.

A CRISPR RNA targeting the *TRAC* locus^4^ was cloned into the BsmBI sites between the U6 promoter and the trans-activating CRISPR RNA (tracrRNA) in the vectors above as described,^23^ and vectors expressing the *TRAC* sgRNA were used in downstream assays, including to measure TCR expression.

### Production of lentiviral stocks

Lentiviral stocks were made by transfection of HEK293T/17 cells (ATCC, Manassas, VA, USA) in a six-well format, pooling 6–10 wells to generate each stock. One day prior to transfection, cells were plated at 5 × 10^5^ cells per well in 2 mL of Dulbecco’s modified Eagle’s medium (DMEM) complete (supplemented with 10% fetal bovine serum (FBS), 2 mM l-glutamine and penicillin/streptomycin/fungizone) such that on the day of transfection, cells were at 50%–60% confluency. On the day of transfection, serum-free DMEM and Mirus LT1 DNA transfection reagent (Mirus Bio, Madison, WI, USA) were equilibrated to room temperature. For each well, 667 ng of a given transfer vector was mixed with 500 ng of the psPAX2 packaging vector (Addgene 12260) and 333 ng of the pMD2.G VSV-G expression vector (Addgene 12259) in 200 μL of serum-free media, before addition of 4.5 μL of transfection reagent with gentle mixing and incubation for 15–30 min at room temperature. The DNA/Mirus mixture was then added to each well in a drop-wise manner.

At 20–22 h post-transfection, media were aspirated and replaced with 1.5 mL of fresh DMEM complete per well. This was done to concentrate viral stocks and to dilute carryover plasmids for downstream analyses.

Two days after transfection, supernatants from multiple wells were pooled, filtered through a 0.2-μm filter, and brought up to a final volume of 35 mL with ice-cold PBS. Diluted supernatants were concentrated 25- to 40-fold by layering over 2 mL of cold 20% sucrose before ultra-centrifugation at 23,000 rpm for 1 h at 4°C. Supernatants were poured off the sucrose pellet, which was subsequently resuspended in 200–250 μL of RPMI 1640 complete medium, resulting in a 25- to 40-fold concentration of viral stock. Pellets were resuspended by gentle pipetting and periodic low-intensity vortexing and left at 4°C overnight before aliquoting and freezing down at −80°C. The concentration of viral stocks was quantified by determining p24 concentration using a p24 ELISA (Advanced BioScience Laboratories, Rockville, MD, USA) on viral supernatants that had been diluted 100,000- to 300,000-fold.

### Isolation and initial activation of primary CD4^+^ and CD8^+^ T cells

Whole blood from healthy donors was obtained from Bloodworks Northwest, and total peripheral blood mononuclear cells (PBMCs) were isolated by purification over Histopaque-1077 (Sigma-Aldrich, St. Louis, MO, USA). Pure populations of CD4^+^ and CD8^+^ T cells were isolated using negative selection (STEMCELL Technologies, Vancouver, BC, Canada). Cells were resuspended at 2–2.5 × 10^6^ cells/mL in 96-well plates with RPMI 1640 complete medium supplemented with 10% FBS, 2 mM l-glutamine, penicillin/streptomycin/fungizone, and 100 U/mL interleukin (IL)-2 (Roche, Basel, Switzerland) and activated with plate-bound anti-CD3 clone UCHT1 at a concentration of 10 μg/mL and free-floating anti-CD28 clone CD28.2 at a concentration of 5 μg/mL (Tonbo Biosciences, San Diego, CA, USA).

### Transduction of cells with lentiviral constructs and titration by flow cytometry

Cells at 1.5–2.5 × 10^6^ cells/mL were transduced at 20–24 h post-activation with the indicated amount of viral inoculum per million cells. Each well was transduced with 10 μL of viral inoculum supplemented with protamine sulfate (Sigma-Aldrich, St. Louis, MO, USA) to a final concentration of 8 μg/mL when added to cells. The inoculum was mixed gently with the cells, which were subsequently spinoculated at 1,100 × *g* for 90 min at 30°C and maintained at 37°C thereafter.

Cells were transduced with 2 and 0.2 ng of p24 per million cells in the case of TOPmChΔW and lentiGuide.mCherry and 20 and 2 ng of p24 per million cells in the case of the various CAR expression vectors. TOPmChΔW and lentiGuide.mCherry-transduced cells were assayed by flow cytometry detection of mCherry expression at 3 days post-transduction, and CAR expression vectors were evaluated at 4 days post-transduction by the detection method detailed below. Gates were determined using mock-transduced cells as negative controls, and transduced cultures displaying 5%–20% positive cells were used to determine transduction titers. Sample flow cytometry plots are included in Figure S2.

### Detection of streptavidin-tagged CAR

Two days post-transduction with TOP-CAR constructs, cells were resuspended to 2–2.5 × 10^6^ cells/mL in RPMI 1640 complete medium supplemented with 100 U/mL recombinant IL-2 (Roche, Basel, Switzerland) and further activated for 3 days with anti-CD2/anti-CD3/anti-CD28 beads (Miltenyi Biotec, Bergisch Gladbach, Germany) at a bead-to-cell ratio of 1:1 and maintained at 1 × 10^6^ cells/mL in RPMI 1640 complete medium supplemented with 100 U/mL of recombinant IL-2 (Roche, Basel, Switzerland).

CAR expression was detected at 4 and 10 days post-transduction with a fluorescein isothiocyanate (FITC)-tagged anti-streptavidin antibody (GenScript, Piscataway, NJ, USA) according to the manufacturer’s protocols. Briefly, 100,000–200,000 cells per transduction were pelleted at 300 × *g* at room temperature for 10 min in a V-bottom plate. Cells were washed once in PBS before incubation for 30 min at 37°C in blocking buffer (3% bovine serum albumin in PBS). Cells were spun down and resuspended in 100 μL of blocking buffer with 1.5 μg/mL antibody and incubated for 1 h in at 37°C. Cells were pelleted and washed once in wash buffer (0.05% Tween 20 in PBS), fixed in 1% paraformaldehyde, and immediately read by flow cytometry.

### CRISPR-Cas9-mediated editing of the *TRAC* locus and detection of loss of the TCR

CRISPR-Cas9 assembly and electroporation was performed largely as described^24^ using a modified guide-swap strategy.^3^ Each CRISPR-Cas9 non-targeting guide RNA complex (cRNP) was pre-assembled by first pre-incubating 1 μL of 160 nM non-targeting CRISPR-RNA with 1 μL of 160 nM tracrRNA (both from Integrated DNA Technologies, Coralville, IA, USA) at 37°C for 30 min. The CRISPR-RNA/tracrRNA complex was then incubated for 15 min at 37°C with 2 μL of a 40 μM SpCas9 protein (Berkeley Macromolecular Facility, Berkeley, CA, USA) for a final ratio of 4:1 RNA:SpCas9. Complexes were frozen down at −80°C until ready for use.

Primary T cells were isolated and stimulated as described above. One day after activation, cells were transduced as described with 20 ng of p24 per million cells in the case of TOPmChΔW and lentiGuide.mCherry or 60 ng of p24 per million cells in the case of TOP-CARΔW. Two days after transduction, 1 million cells per electroporation were pelleted at 100 × *g* for 10 min and washed once with PBS. Cells were resuspended with 20 μL of P3 primary cell Nucleofector solution (Lonza, Basel, Switzerland) and mixed gently with 4 μL of the cRNP complexes described above. Cells and complexes were incubated together for ~5 min at room temperature before electroporation in a 16-well Nucleocuvette in an Amaxa Nucleofector (Lonza, Basel, Switzerland) using pulse code EH-115. Cells were supplemented with 80 μL of RPMI 1640 complete medium and allowed to recover for 2 h at 37°C before plating at 2.5 × 10^6^ cells/mL at a 1:1 ratio with activation beads (Miltenyi Biotec, Bergisch Gladbach, Germany) in RPMI 1640 complete medium with 100 U/mL recombinant IL-2 (Roche, Basel, Switzerland). Two days later, each electroporation was supplemented with an additional 200 μL of RPMI 1640 complete medium with 100 U/mL recombinant IL-2 (Roche, Basel, Switzerland). Four days after electroporation, 100,000–200,000 cells were stained for CAR expression as described above, as well as TCR expression using a phycoerythrin (PE)-conjugated antibody targeting CD3 (BD Biosciences, San Jose, CA, USA) as has been described previously^4^ or with a FITC-conjugated antibody targeting CD3 (BD Biosciences, San Jose, CA, USA) in the case of the cells transduced with TOPmChΔW or lentiGuide.mCherry.

### Detection of packaging of lentiviral genomes with droplet digital PCR

Lentiviral stocks were made in triplicate transfections with different vector plasmid preparations as described above. Supernatants were filtered through a 0.2-μm filter, but were not concentrated prior to freezing at −80°C. The p24 concentrations of the frozen aliquots were determined as described above on supernatants that had been diluted 10,000- to 30,000-fold in DMEM.

Viral RNA was isolated using a QIAGEN viral RNA kit (QIAGEN, Hilden, Germany) and subjected to DNase I treatment (Roche, Basel, Switzerland) for 2 h at 37°C, followed by a 5-min inactivation at 75°C. Viral RNA was reverse transcribed into cDNA using random hexamers and was diluted for amplification by droplet digital PCR (ddPCR). Ultimately the equivalent of 0.01 or 0.003 μL of cDNA was tested because it provided a quantifiable range of template. Controls lacking reverse transcriptase were used to determine the amount of background plasmid contamination, which was below detection of the assay. Values were reported as “normalized genome copies,” which represents the number of ddPCR-positive droplets per μL of cDNA divided by the concentration of p24 in ng/mL.

The amplification primers used were as follows: forward, 5′-GACTAGCGGAGGCTAGAAGGAGAGA-3′; reverse, 5′-CTAATTCTCCCCCGCTTAATAYTGACG-3′; with probe 5′-AT+G+GGT+GC+GAGA-3′. These primers amplify a region spanning a portion of the 5′ LTR and early HIV-1 *gag* sequence that is common to all constructs assayed.

## Supplemental Information

Document S1. Figures S1–S3

Document S2. Article plus supplemental information
