# Supplementary material for: The TOP vector: a new high-titer lentiviral construct for delivery of sgRNAs and transgenes to primary T cells
Source: Mol Ther Methods Clin Dev. 2020 Oct 27;20:30–8. doi: 10.1016/j.omtm.2020.10.020 (PMC7732963; doi:10.1016/j.omtm.2020.10.020)
Supplement: Document S1. Figures S1–S3 [file mmc1.pdf]

OMTM, Volume *20*

Supplemental Information

The TOP vector: a new high-titer lentiviral construct for delivery of sgRNAs and transgenes to primary T cells

Daryl Humes, Stephanie Rainwater, and Julie Overbaugh

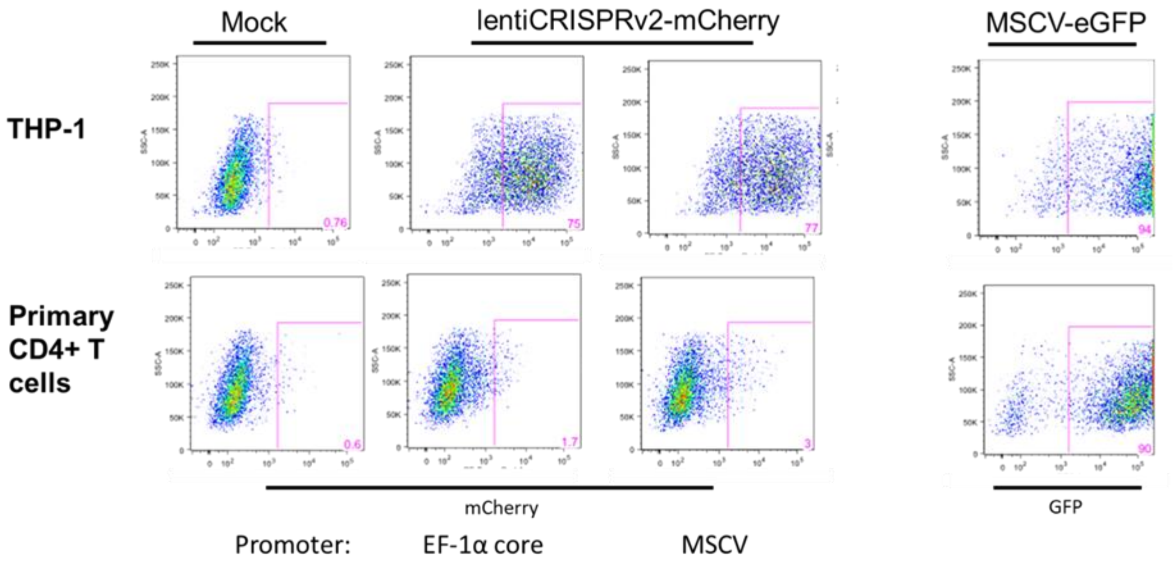

S1. Transduction of THP-1 cells and primary CD4+ T cells with SpCas9:mCherry driven by different promoters.

The indicated cell types were transduced with equivalent saturating amounts of lentiCRISPRv2-mCherry (AddGene 99154), in which SpCas9 is linked to mCherry expression by a P2A linker and is driven by the EF-1α core promoter; or by an identical construct in which SpCas9:mCherry expression is driven by the MSCV promoter. MSCV-eGFP, in which eGFP expression is driven by the MSCV promoter was included to show that primary CD4+ T cells were transducible in this experiment.

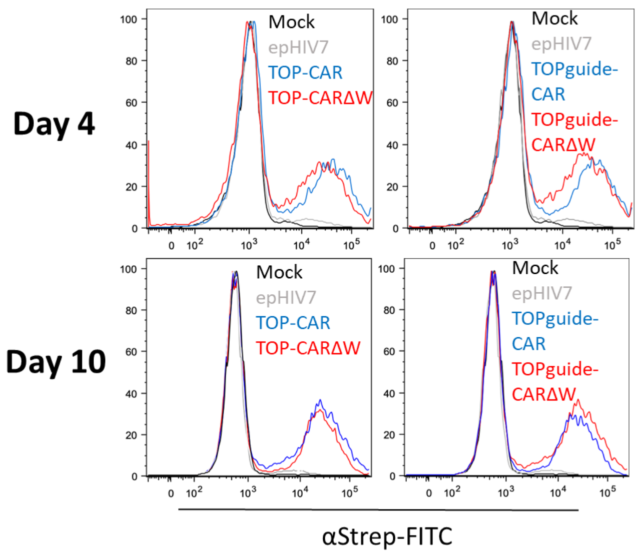

S2. Sample flow cytometry histograms showing relative CAR expression of different vectors.

Shown are cells transduced with 20 ng p24 per million cells of a given vector at the indicated days post-transduction. For ease of interpretation histograms have been separated, plots on the left show TOP-CAR (blue) and TOP-CARΔW (red) and plots on the right show TOPguide-CAR (blue) and TOPguide-CARΔW (red) compared to the same mock transduced (black) and epHIV7-CAR (grey) transduced cells. The plots shown were derived from transduced CD8+ T cells from the donor corresponding to the green data point in Figure 3 b).

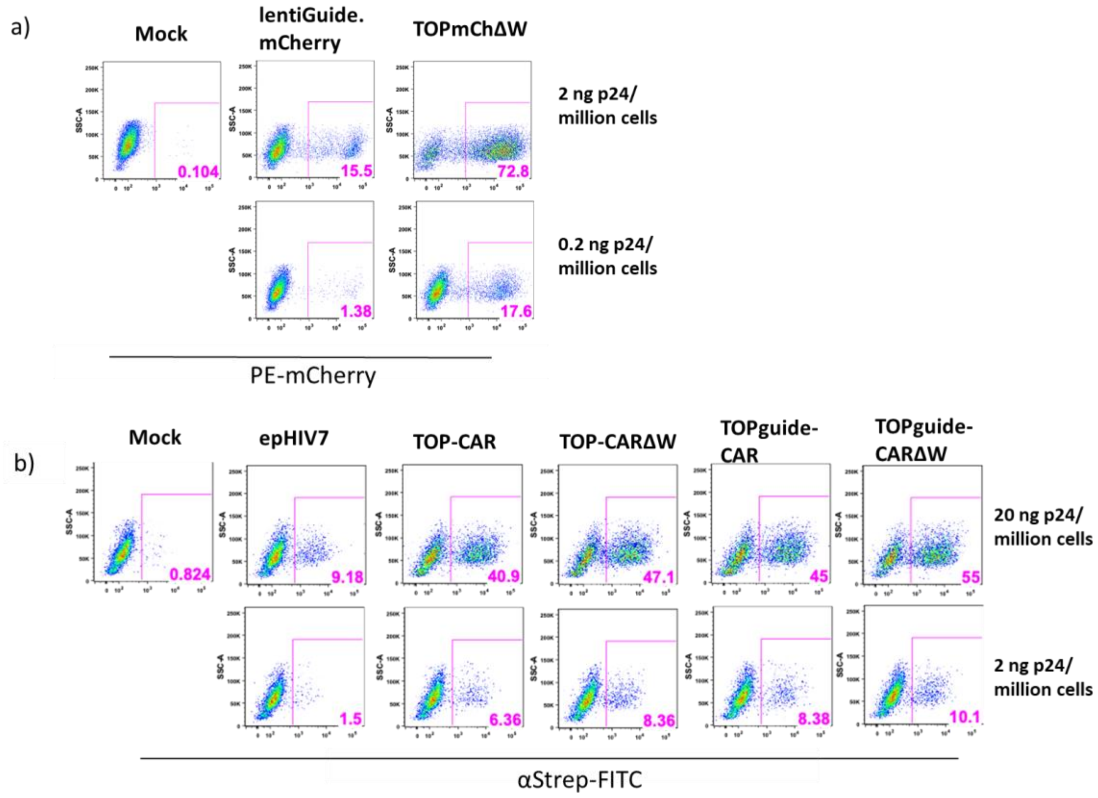

S3. Sample flow cytometry plots of transduced cells.

Cells were transduced with the indicated amounts of lentiGuide.mCherry and TOPmChΔW, **a)**, or CAR expressing vectors, **b)**, and assayed by flow cytometry as described in the Materials and Methods. The plots shown were derived from transduced CD4+ T cells from the donor corresponding to the black data points in Figures 1 b) and 3 a).
